# Circulating Biomarkers to Identify Responders in Cardiac Cell therapy

**DOI:** 10.1038/s41598-017-04801-7

**Published:** 2017-06-30

**Authors:** Jesse V. Jokerst, Nicholas Cauwenberghs, Tatiana Kuznetsova, Francois Haddad, Timothy Sweeney, Jiayi Hou, Yael Rosenberg-Hasson, Eric Zhao, Robert Schutt, Roberto Bolli, Jay H. Traverse, Carl J. Pepine, Timothy D. Henry, Ivonne H. Schulman, Lem Moyé, Doris A. Taylor, Phillip C. Yang

**Affiliations:** 1 Department of NanoEngineering, University of California, San Diego, USA; 2 Research Unit of Hypertension and Cardiovascular Epidemiology, Department of Cardiovascular Sciences, University of Leuven, Leuven, Belgium; 3 Department of Medicine, Stanford University, Stanford, USA; 4 Clinical and Translational Research Institute, University of California, San Diego, USA; 5 Houston Methodist Hospital, Houston, USA; 6 School of Medicine, University of Louisville, Louisville, USA; 7 Minneapolis Heart Institute Foundation at Abbott Northwestern Hospital, Minneapolis, USA; 8 University of Florida College of Medicine, Gainesville, USA; 9 Cedars-Sinai Heart Institute, Los Angeles, USA; 10 Interdisciplinary Stem Cell Institute, University of Miami Miller School of Medicine, Miami, USA; 11 University of Texas School of Public Health, University of Texas Health Science Center, Houston, USA; 12 Texas Heart Institute, CHI St. Luke’s Health Baylor College of Medicine Medical Center, Houston, USA

## Abstract

Bone marrow mononuclear cell (BM-MNC) therapy in ST-elevation acute myocardial infarction (STEMI) has no biological inclusion criteria. Here, we analyzed 63 biomarkers and cytokines in baseline plasma samples from 77 STEMI patients treated with BM-MNCs in the TIME and Late-TIME trials as well as 61 STEMI patients treated with placebo. Response to cell therapy was defined by changes in left ventricular ejection fraction, systolic/diastolic volumes, and wall motion indexes. We investigated the clinical value of circulating proteins in outcome prediction using significance testing, partial least squares discriminant analysis, and receiver operating characteristic (ROC) analysis. Responders had higher biomarker levels (76–94% elevated) than non-responders. Several biomarkers had values that differed significantly (P < 0.05) between responders and non-responders including stem cell factor, platelet-derived growth factor, and interleukin-15. We then used these lead candidates for ROC analysis and found multiple biomarkers with values areas under the curve >0.70 including interleukin 15. These biomarkers were not involved in the placebo-treated subjects suggesting that they may have predictive power. We conclude that plasma profiling after STEMI may help identify patients with a greater likelihood of response to cell-based treatment. Prospective trials are needed to assess the predictive value of the circulating biomarkers.

## Introduction

Cell-based cardiac therapy has produced encouraging but inconsistent results in treating ischemic heart disease^1–3^. One current hypothesis is that its efficacy is limited because of poor engraftment into the heart after delivery and rapid cell death^2,4^. Schemes to increase cell survival and engraftment include, among many other, support scaffolds^5^ and co-delivery of pro-survival agents^6,7^. However, these approaches do not address a deeper issue: within any patient population that there is likely a wide variation in the effectiveness of stem cells. Current inclusion criteria for ST-elevation myocardial infarction (STEMI) cell therapy trials^8^ largely use cardiac function indexes such as left ventricular ejection fraction (LVEF). Yet, these indices only reflect the extent of cardiac damage after reperfusion and provide guideline for subsequent medical therapy. They offer no molecular level information to better identify patients who would benefit from cell therapy. In addition to current selection criteria, circulating proteins might have additional value in predicting individual response to cell therapy. Such biomarkers and cytokines might indicate a robust microenvironment capable of promoting cell survival and functional recovery. Just as biomarkers for graft-versus-host disease following allogeneic hematopoietic cell transplantation have the promise to predict response^9,10^, we hypothesized that this approach might identify positive responders in cell-based therapy for STEMI patients.

Candidate biomarkers include circulating proteins such as stem cell factor (SCF), stromal cell derived factor (SDF1), and colony stimulating factors^11^, which are known to have a role in myocardial regeneration. Similarly, biomarkers involved in cell growth (e.g. vascular endothelial growth factor; VEGF)^12^, repair (tumor necrosis factor α; TNFα)^12^, adhesion (vascular cell adhesion molecule; VCAM1)^13^, and inflammation (interleukins and chemokines) might predict the outcome of cell therapy.

In this study, we measured 63 circulating proteins in baseline serum samples obtained from reperfused STEMI patients (N = 138) who were then treated with autologous BM-MNCs (150 × 10^6^) or placebo. The data were obtained from both the TIME^14^ and LateTIME trials^15^ conducted by the Cardiovascular Cell Therapy Research Network. While these trials did not show an aggregate benefit to therapy relative to placebo controls, there were “responders”, i.e., patients in whom cardiac function improved, within both the cell-treated and placebo-treated cohorts. Our goal here was to profile the serum of responders and non-responders in both cohorts and identify the differences in biomarker profiles, which correlated positively with a response to cell therapy.

To the best of our knowledge, this is the first report to profile a set of circulating biomarkers and investigate their clinical value in predicting the response to cardiac cell therapy after STEMI. These biochemical signatures may improve the selection of STEMI patients and personalize cell therapy.

## Methods

### Enrollment and Specimens

Banked plasma samples were obtained from the TIME (clinicaltrials.gov no. NCT00684021) and Late-TIME trial (clinicaltrials.gov No. NCT00684060), which were conducted between July 2008 and November 2011. Both studies assessed left ventricle function at baseline and six months after intracoronary delivery of BM-MNCs after STEMI. BM-MNC administration was performed 3 and 7 days (TIME) or 14–21 days (Late-TIME) after reperfusion. The primary inclusion criterion was a left ventricle ejection fraction (LVEF) <45% as assessed by echocardiography. Specimens in TIME were collected within a week of the myocardial infarction. Specimens in LateTIME were in general 2–3 weeks after the infarction. Patients with previous bypass surgery or prior STEMI with residual left ventricular dysfunction were excluded. Blood samples were collected after reperfusion but prior to administration of cells in tubes containing EDTA as an anticoagulant. Blood samples were centrifuged and plasma was decanted and stored at −80 °C. All samples were collected after subjects provided written informed consent. This study received Institutional Review Board approval by the University of Texas IRB. All patients provided written informed consent following broad discussions of the risks, benefits, and alternatives of the TIME and LateTIME trials. All methods were performed in accordance with the relevant guidelines and regulations and the Declaration of Helsinki.

### Data Analysis

Patients were classified as either responders or non-responders based on the original outcome data. For example, a patient with a positive ΔEF was considered a responder as was a patient with a decreased ΔEDVI. In addition to the five metrics above, we also included a subset of high ΔEF responders. These were patients with a ΔEF of >5 (absolute increase in ΔEF >5). Next, for each biomarker, a mean and two-sided student’s t-test were calculated using the raw mean fluorescent intensity (MFI) data. This MFI approach has utility when samples have low levels of biomarker because interpretation with a sigmoidal calibration curve can suppress subtle differences^16^.

We compared the mean biomarker levels between the cell-treated and placebo-treated cohort, and calculated P values (2-tailed, homoscedastic) to determine the significance of these changes. These significant biomarkers as well as biomarkers with mean values of more than 2.0-fold different between responders and non-responders were characterized with receiver operating characteristic (ROC) curve; an area under the curve (AUC) and an associated P value was calculated with GraphPad Prism. P values < 0.05 were considered statistically significant.

### Partial Least Square Discriminant Analysis (PLS-DA)

PLS-DA analysis used SAS software, version 9.3, and JMP Genomics, version 6.0 (SAS Institute, Cary, North Carolina). Samples were adjusted for age, sex, and BMI. Using PLS-DA, we assessed the relation between circulating biomarkers and absolute or relative changes in ejection fraction (EF), end diastolic volume index (EDVI), end systolic volume index (ESVI), infarct zone wall motion (INZ), or border zone wall motion (BDZ) analyzed as continuous variables. In categorical analysis, we identified a pattern of cytokines associated with response to stem cell therapy (responders versus non-responders). PLS-DA creates several linear combinations (latent factors) of the log-transformed predictors to maximize the covariance between the predictors and the outcome variables^16,17^. We assumed that the number of latent factors was not significantly different from the model with the minimum predicted residuals sum of squares (PRESS) value^18^. These latent variables were then used in the discrimination analysis instead of the original individual predictors (biomarkers).

The importance of each cytokine in the construction of the latent variables is assessed from the variables importance for projection (VIP) scores as proposed by Wold^16,19^. In both continuous and categorical analysis, cytokines with a VIP >1.5 were considered influential. The distributions of all cytokines were analyzed by transformation to the common logarithm. The VIP score was calculated as the sum of the squared correlations between the latent factors and the biomarker, weighted for the percentage of variation explained by the latent factors in the model. Finally, V-plots were constructed to illustrate the importance of each biomarker in the model (VIP, x axis) in relation to the coefficient size of each biomarker (y axis). These V plots illustrate the contribution of all biomarkers to the model used to explain outcome as well as their effect on the outcome.

### Net Reclassification Index (NRI)

We also performed NRI analysis in the cell-treated patients to determine whether the selected biomarkers better reclassified the treated patients by response. The default classifier consisted of age and sex, whereas the new classifier additionally included the latent factor(s) derived from the informative biomarkers and related to the treatment response (dEF, dEDV, etc.)

## Results

### Clinical Features of the Study Population

Detailed descriptions of the trial participants are available in the TIME^14^ and Late-TIME^15^ manuscripts. The majority of the participants were male (73%), white (78%), and hyperlipidemic (70%). Table 1 lists the clinical and biochemical characteristics of the responders (ΔEF > 0, n = 49) and non-responders (ΔEF ≤ 0, n = 29) in the cell treated group. We noted no significant difference in clinical, demographic and biochemical characteristics between the two subgroups (P ≥ 0.09; Table 1). We repeated the comparison between responders who received BM-MNCs and responders who received placebo. Just as in the BM-MNC group, placebo “responders” has a positive outcome (+ΔEF, −ΔEDVI, −ΔESVI, +ΔINZ, or +ΔINZ). These were patients in TIME or LateTIME who received placebo and had a positive outcome (response). The only significant difference between placebo responders and cell treatment responders was in baseline heart rate; (72.4 ± 19.9 in the treated cohort; 81.8 ± 22.2 bpm in the placebo cohort; P = 0.035).

**Table 1:** Clinical features of responders (ΔEF > 0) and non-responders (ΔEF > 0) treated with either cell BM-MNCs or placebo. Values are mean (±SD) or number of subjects (%).

	Cell Treated			Placebo		
	Non-Responders	Responders	P values	Non-Responders	Responders	P values
	(n = 28)	(N = 49)		(n = 18)	(N = 42)	
Age (y)	59.8 ± 11.7	56.1 ± 11.4	0.17	54.4 ± 11.2	55.9 ± 11.2	0.64
Gender (male)	23 (79%)	41 (84%)	0.63	14 (78%)	39 (93%)	0.099
BMI	28.8 ± 4.7	28.9 ± 5.8	0.96	30.1 ± 5.8	29.1 ± 4.6	0.48
Hgb (g/dL)	13.8 ± 1.6	13.6 ± 1.3	0.60	12.8 ± 1.6	13.5 ± 1.9	0.21
hsCRP	22.7 ± 36.5	23.6 ± 27.2	0.90	35.1 ± 30.8	27.0 ± 31.5	0.4
CKMB (U/mL)	341 ± 269	271 ± 172	0.24	319 ± 261	250 ± 179	0.29
HR (bpm)	79.9 ± 18.0	72.4 ± 19.9	0.10	87.0 ± 17.4	81.8 ± 22.2	0.38
EF (%)	48.9 ± 11.9	44.3 ± 10.9	0.09	46.0 ± 10.2	43.0 ± 9.4	0.28
Hyperlipidemia	21 (72%)	34 (69%)	0.78	13 (72%)	29 (69%)	0.81
Angina	6 (20%)	12 (24%)	0.70	3 (17%)	6 (14%)	0.82
Diabetes	6 (20%)	5 (10%)	0.20	4 (22%)	8 (19%)	0.78
HTN	17 (59%)	28 (57%)	0.90	11 (61%)	25 (60%)	0.91
Smoking	15 (51%)	32 (65%)	0.24	11 (61%)	26 (62%)	0.95

### Luminex Assay Quality Control

**Figure 1:**
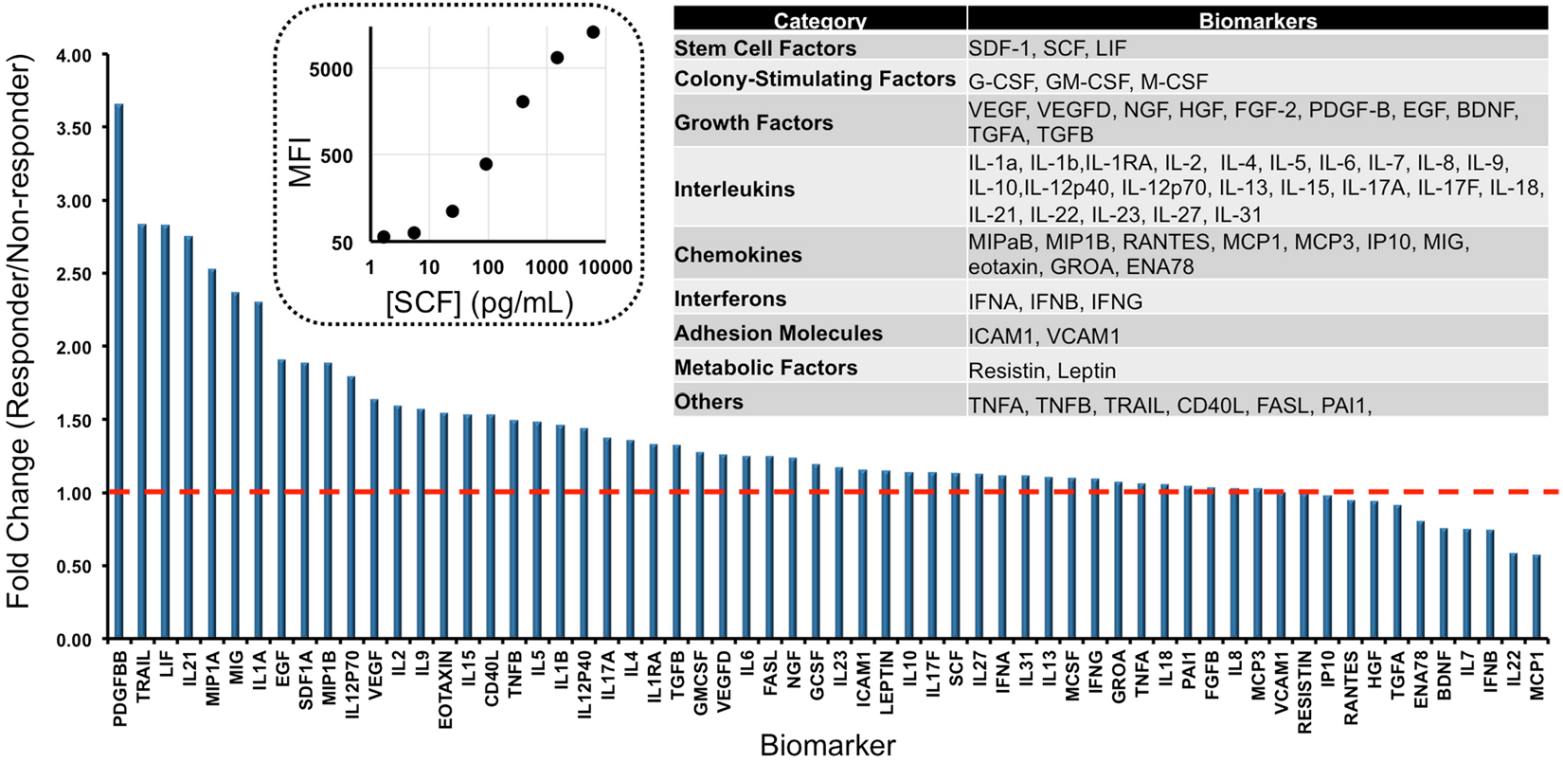
Biomarkers used in the study. Fold change of responders (ΔEF > 0) versus non-responders (ΔEF ≤ 0) is demonstrated. Dashed inset graph shows the representative calibration curve for stem cell factor. Right upper table shows the broad classes of the biomarkers used in present study. Abbreviations. BDNF: brain-derived neurotrophic factor; CXCL: chemokine; EGF: epidermal growth factor; ENA78: CXCL5; FASL: fatty acid synthase ligand; FGF2: fibroblast growth factor; G-CSF: granulocyte colony-stimulating factor; GM-CSF; granulocyte macrophage colony-stimulating factor; GROA: CXCL1; HGF: human growth factor; ICAM: intercellular adhesion molecule; IL: interleukin; IP10: interferon gamma-induced protein (CXCL10); LIF: leukemia inhibitory factor; MCP: monocyte chemoattractant protein; M-CSF: macrophage colony-stimulating factor; MIG: CXCL9; MIP: macrophage inflammatory protein; NGF: nerve growth factor; PAI1: plasminogen activator inhibitor; PDGF-B: platelet derived growth factor; RANTES: regulated on activation normal T cell expressed and secreted (aka CCL5); SCF: stem cell factor; SDF1: stromal cell derived factor; TGFA: transforming growth factor α; TNF: tumor necrosis factor; TRAIL: TNF-related apoptosis-inducing ligand; VCAM: vascular adhesion molecule; VEGF: vascular endothelial growth factor.

We validated the assays using standard curves and calibration samples for every biomarker according to standard Luminex protocols (Fig. 1)^16,20,21^. The inset and caption of Fig. 1 lists the complete biomarker cohort and describes their potential role in cardiac repair. The coefficient of variation (CV) of the assays were below 12% except for IL-22 (55.1%), IL-23 (23.3%), MCP1 (27.7%), and RANTES (15.3%). The reason for the large CV values is that these assays had low bead counts (<~25 beads). While these results were included in subsequent analysis, only RANTES showed any correlation with outcome or predictive power. Thus, the RANTES data should be interpreted with caution.

### Baseline Biomarker Levels in Responders versus Non-responders

Next, the mean of the 63 biomarkers measured in the baseline samples were compared between the responders and non-responders using the raw data (MFI). We used these mean values to compare the fold-change in biomarker levels between responders and non-responders (Fig. 1) in both the BM-MNC-treated and placebo cohorts.

The fold-change in the ΔEF cohort is measured by averaging the biomarker value of responders normalized to the average biomarker value of non-responders in the treated cohort (Fig. 1). Assuming an equal and random distribution, one would expect approximately the same number above and below a value of unity (red line), but this is not the case in the BM-MNC-treated group. The majority (≥76%) of the 63 baseline biomarkers were increased in BM-MNC-treated responders versus non-responders regardless of the response metric. For the ΔEF response metric, 50 of the 63 biomarkers were elevated (79%) in responders at baseline (Fig. 1), and other outcome metrics followed a similar trend: ΔEDVI (59/63 biomarkers or 94%); ΔESVI (48/63, 76%); ΔINZ (52/63, 83%); ΔBDZ (48/63, 76%); and ΔEF > 5 (52/63, 83%). In placebo-treated subjects, ≤76% of the biomarkers were elevated in responders: ΔEF (48/63; 76%); ΔEDVI (34/63; 54%); ΔESVI (39/63, 62%); ΔINZ (26/63, 41%); ΔBDZ (32/63, 51%); and ΔEF > 5 (28/63, 44%).

**Figure 2:**
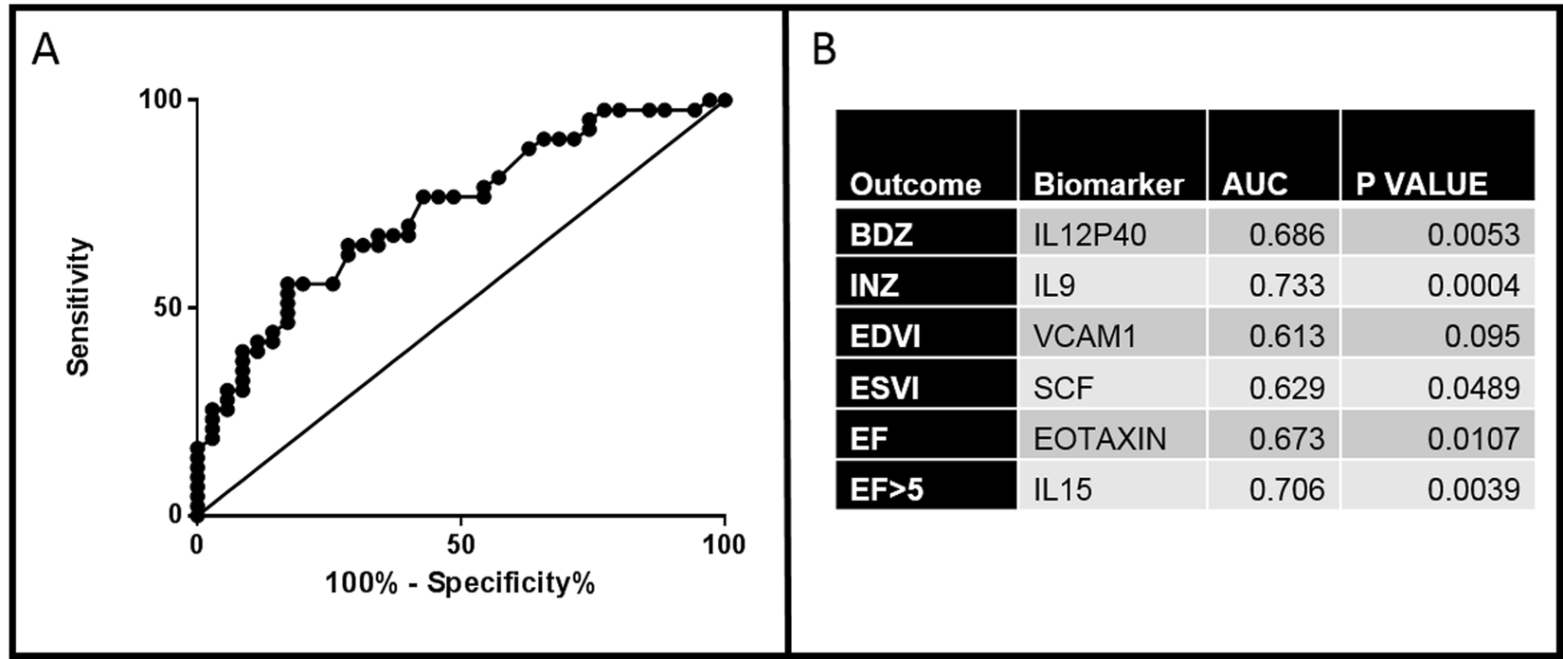
(**A)**. Representative ROC for IL9 with the ΔINZ metric gives an AUC of 0.73 (P < 0.001). Biomarkers with the highest AUC value for each type of outcome metric are shown in panel B.

In many of the highest fold-changes (e.g., PDGFBB in Fig. 1), the high fold-change is largely due to only one or two outliers. Biomarkers with significant differences between the two patient cohorts as well as significant values for both BM-MNC-treated and placebo subjects are presented in Table 2. Although these values did not achieve significance when analyzed with a Bonferroni correction, our goal here is to identify new relationships that require confirmation rather than generalize our results to the population at large. The biomarker with the highest AUC value for each type of outcome metric is reported as well as a representative ROC curve for IL9 (Fig. 2**)**. Multiple biomarkers had AUC values over 0.60.

**Table 2:** Biomarkers with a p value <0.05 between responders vs. non-responders for different outcome metrics in both BM-MNC-treated and placebo cohorts.

	BM-MNC-Treated Subjects	Placebo Subjects
ΔEF	IL27 (0.05); Eotaxin (0.009);	IL1B (0.044)
ΔESVI	IL27 (0.049); IL5 (0.042); NGF (0.045);	IL15 (0.0040); IL18 (0.048)
ΔEDVI	VCAM1 (0.04)	None
ΔINZ	FASL (0.004); IL4 (0.04); IL8 (0.003); IL31 (0.005); IL9 (0.016);	IL1B (0.012)
ΔBDZ	Eotaxin (0.021); IL9 (0.048); IL12p40 (0.047)	HGF (0.040); VEGFD (0.017)
ΔEF > 5	Eotaxin (0.029); IL1RA (0.042); IL12p40 (0.025)	None

### Discrimination Analysis using PLS-DA for Identification of Important Biomarkers in Stem Cell Response

**Figure 3:**
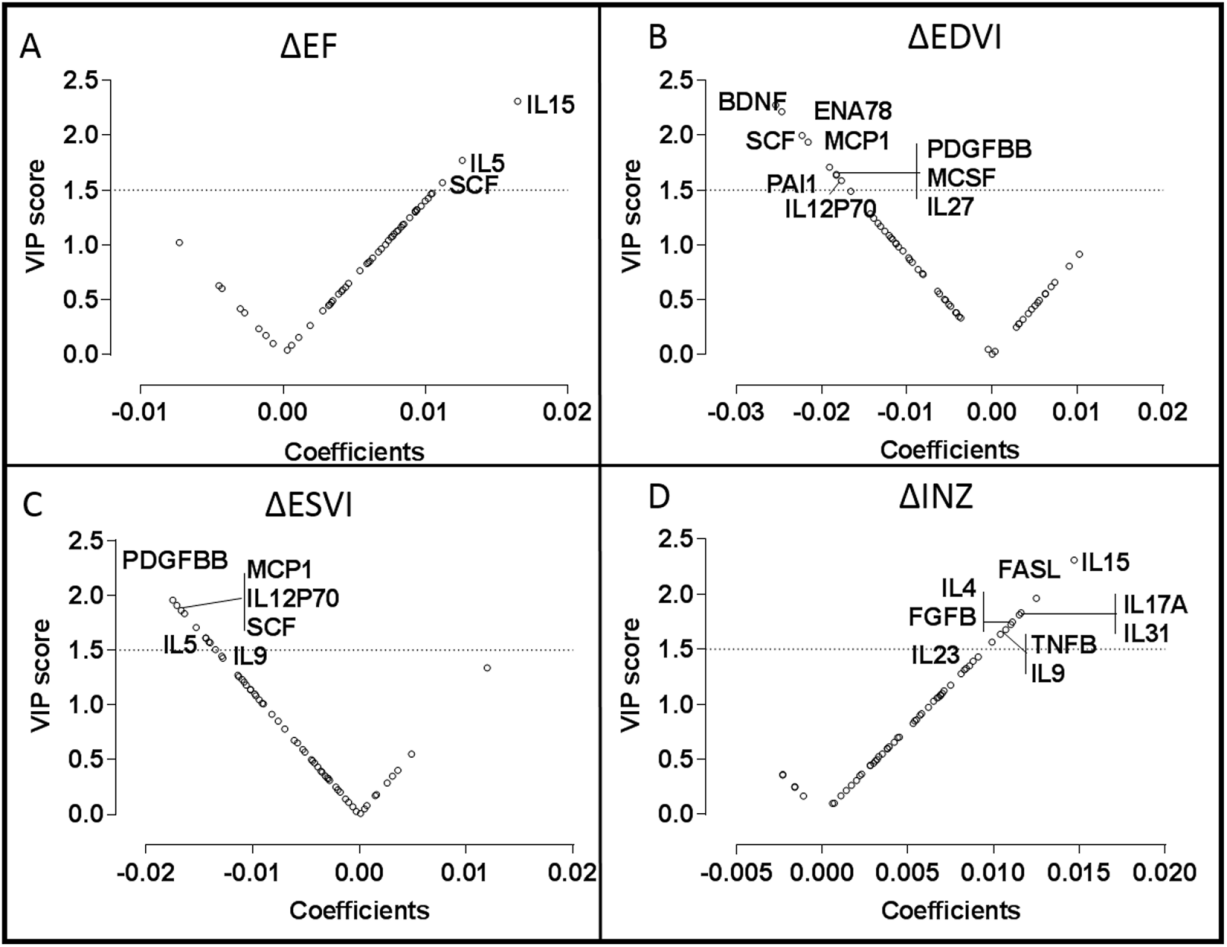
V-plots for continuous PLS-DA models generated with extracted VIP and correlation coefficient values for the BMC-treated subjects. Biomarkers with high VIP (>1.5) and coefficient values are named. V-plots for continuous change in in (**A**) EF, (**B**) EDVI, (**C**) ESVI, and (**D**) INZ.

**Figure 4:**
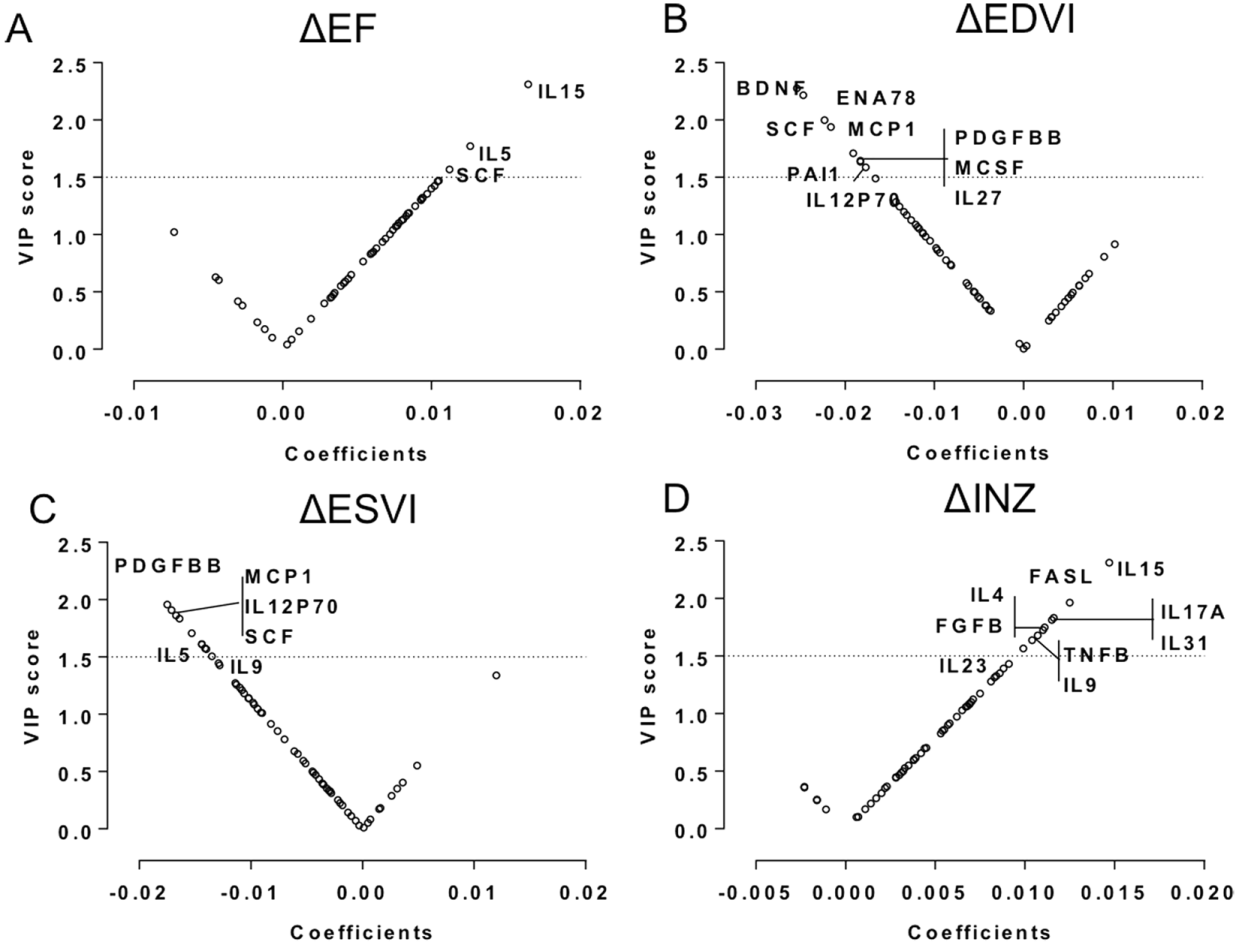
V-plots for categorical PLS-DA models generated with extracted VIP and correlation coefficient values for BMC-treated subjects. Biomarkers with high VIP (>1.5) and coefficient values are named. V-plots for a favorable change in (**A**) EF, (**B**) EDVI, (**C**) ESVI, (**D**) INZ, (**E**) BDZ, and (**F**) EF, EDVI + ESVI.

The V plots (Fig. 3 for continuous, Fig. 4 for categorical) show a cytokine variable importance in projection (VIP) score in modeling of latent factors versus the centered and scaled correlation coefficients between cytokine and outcome. Cytokines with higher VIP values (VIP > 1.5) were more important for class discrimination. Cytokines with the highest VIP values and positive correlation with respect to the outcome are present in the upper far right quadrant while cytokines with the highest VIP values and negative correlation with respect to the outcome are placed in the upper far left quadrant.

Biomarkers with elevated VIP values (VIP > 1.5) in continuous and categorical PLS-DA analysis are presented in Supplementary Table 1 and Table 2, respectively. Overall, we observed that a higher IL15, IL5, and SCF were related to more favorable changes in several LV indexes in the BM-MNC-treated group: increase in ΔEF, ΔINZ and ΔBDZ and decrease in ΔEDVI and ΔESVI (Supplementary Table 3; Figs 3 and 4). Moreover, in categorical analyses, a higher IL15 was related to a greater increase in ΔEF and ΔBDZ after cell therapy, whereas a higher SCF significantly predicted a more favorable response in ΔEF and LV volume indexes (Supplementary Table 1; Fig. 3). Furthermore, in both continuous and categorical PLS analyses, MCP1, IL27, and IL12p70 were predictive of the decrease in EDVI and ESVI after cell therapy (Supplementary Tables 1 and 2; Figs 3 and 4). We additionally defined a subset of patients presenting a trio of favorable responses, i.e. ΔEF > 0, ΔESVI < 0, and ΔEDVI < 0 (n = 18; Supplementary Table 4, Fig. 4F). In these ‘triple-responders’, biomarkers with positive coefficients and high VIP included: VCAM1 (VIP, 1.51), SCF (2.17), PDGFBB (1.53), MCSF (1.78), IL27 (1.80), IL13 (1.63), IL12p70 (2.04), and BDNF (1.71).

PLS-DA analysis was repeated in placebo patients (Supplementary Tables 3 and 4) and identified several biomarkers that were predictive of changes in outcome. These biomarkers correlated to improved outcome independent of cell therapy. Importantly, the biomarkers identified in the placebo group did not correspond to those in the treatment group (IL15, IL5, SCF, etc.).

Finally, we compared the biomarker levels in placebo responders and BM-MNC responders to determine if there were any inherent differences in baseline physiology. Here, we only used the LVEF metric. None of the lead biomarkers identified above were different between the two sets of responders. Biomarkers that did have a significant difference included: MCP3, IL18, PAI1, TNFa, IFN-G, RANTES, IL13, and IL6.

### Net Reclassification Index (NRI)

The NRI is only statistically significant for dEF (Table 3), but the *P* value for NRI is heavily dependent on the number of subjects. As in most NRI analyses, we should not overestimate the importance of this NRI *P* value and instead focus on the absolute NRI. Absolute NRI was high (>0.20) for dEF, dINZ, dBDZ and the triple responders, indicating a strong difference in the proportion of patients reclassified by the set of biomarkers selected by PLS between the two sets of non-responder & responder. In short, it thus might be clinically relevant to assess this set of biomarkers for prediction of response to SC treatment.

**Table 3:** Net Reclassification Index (NRI) Analysis for PLS Selected Biomarkers in SC Treated Patients.

Therapy response	NRI (95% CI)	NRI *P* value	Event *P* value	Non-Event *P* value
ΔEF > 0% (n = 49)	0.48 (0.05 to 0.90)	0.035	0.32	0.056
ΔEDVI < 0 (n = 33)	0.030 (−0.41 to 0.47)	0.90	0.67	0.60
ΔESVI < 0 (n = 42)	0.19 (−0.24 to 0.63)	0.39	0.89	0.22
ΔINZ > 0 (n = 43)	0.25 (−0.18 to 0.68)	0.26	0.89	0.12
ΔBDZ > 0 (n = 44)	0.35 (−0.08 to 0.79)	0.12	0.32	0.22
Triple response (n = 18)	0.33 (−0.04 to 0.70)	0.14	0.032	0.0003

## Discussion

We identified significant differences in biomarker concentrations between cell therapy responders and non-responders that changed as a function of the different outcome metrics. This finding was attributed to the multiple metrics, which were used to classify different patient response. One consistent finding, however, was the up-regulation of all biomarkers in the responders compared to the non-responders. While increased levels of cytokines predict worse outcomes in hematopoietic cell therapy^22^, the potential clinical role of the baseline measurement of these cytokines in cell therapy for heart disease may enable a personalized approach to predict responders.

Myocardial infarction results in an inflammatory cascade as the body removes dead cells and matrix debris from the necrotic region while also activating repair mechanisms. Thus, we suspected that the patients in the TIME trial would have higher cytokine levels because they were enrolled at an earlier time point after the onset of STEMI. However, of the 63 biomarkers, 36 were higher in the Late-TIME patients and 27 were higher in the TIME subjects. Only two—IL1RA and IL12P40—showed a significant difference between the two trials. While prolonged periods of inflammation produced worse outcomes and decreased cardiac function^23^, it is possible that elevated levels of cytokines attracted circulating endogenous stem cells, activated endogenous resident stem cells^24^ or initiated self-repair response to complement the cell therapy. Thus, patients with an overall elevated pool of these biomarkers (at least during this baseline measurement) might be more receptive to endogenous repair.

Different biomarkers were implicated in different analysis methods and different outcome metrics. The previously reported factors implicated in cardiac repair, e.g., VEGF, SDF1, and GSCF^25,26^ did not have a pronounced effect in this study. The most frequent biomarkers were SCF and IL15, which were implicated in all analysis methods. SCF had an AUC of 0.63 (ROC P = 0.049) and IL15 had an AUC of 0.70. VCAM-1 was implicated with simple t-testing but ROC analysis did not confirm this finding. Others have suggested that VCAM-1 was a poor predictor of heart disease outcomes^27^. Eotaxin, an eosinophil chemotactic protein, was implicated in multiple outcome metrics^28^. PDGFBB and MIP1 were implicated in stem cell migration and their elevated levels in the responder suggested recruitment of endogenous stem cells^29,30^. A previous study of MCP1, RANTES, and MIP1a showed that the patients with severe left ventricular dysfunction had elevated levels of these markers one month after STEMI^31^.

The PLS-DA analysis identified candidate biomarkers that were consistently up-regulated in patients who responded via multiple outcome metrics. These highly cross-related markers included SCF, MCSF, and IL12p70, which could have roles in either priming the stem cell niche or supporting the implanted stem cells. MCSF and IL12p70 are implicated in the activation of monocytes and T-cells, respectively, and thus may have a role in immune clearance of necrotic tissue; SCF supports stem cell recruitment and activation. SCF, the ligand for c-Kit, was reported to activate multiple signaling pathways including RAS/ERK, PI3-Kinase, Src kinase, and Janus kinase/signal transducer, the activator of JAK/STAT transcription pathways^32^.The JAK/STAT pathway can mobilize mesenchymal stem cells and may help maintain cardiac function^33^ after cell therapy in this subset of patients. In one study, overexpression of SCF by cardiomyocytes was shown to promote stem cell migration and improve outcome after MI^34^, and thus patients with elevated levels of SCF might be more receptive to cell therapy.

IL-15 was highly implicated with multiple outcome metrics. It has been reported to activate natural killer (T) cell proliferation and hematopoiesis^35,36^. Rodent studies showed an anti-apoptotic role for IL-15 with anti-tumor activity^37^. Elevated IL-15 might protect stem cells and encourage proliferation. A recent study of showed that IL-15 and MCP-1 indicate myocardial response to ischemic insult in the early phase after MI via an inflammatory response^38^. Other cytokines like IL-8 and ENA78 correlated with improved outcomes (Figs 3 and 4). These chemokines contain the ELR motif (such as IL-8) and can induce neutrophil infiltration to exert angiogenic effects^39^.

One limitation of this study is its retrospective design. It was impossible to do biomarker profiling before therapy due to the retrospective study design, however we compared all treated subjects and placebo subjects (ignoring responders versus responders) to see if there were any differences in biomarkers levels. The following biomarkers had a significant (p < 0.05, simple t-test) difference: TNFA, IFNG, PAI1, IL18, IFNA, IL6, NGF, MCP3, RANTES, EOTAXIN, IL17A, IL4, IL10, IL22, GMCSF, BDNF, IL12P40, IL1RA, GCSF, and MCSF. However, none of these biomarkers were implicated as predictive in our PLS-DA analysis. Importantly, there was no statistically significant difference in the placebo and treatment cohort for IL-15, SCF, or PDGFBB, which were the most predictive biomarkers.

Future prospective studies are needed to confirm the panel of biomarkers identified in our study as predictors of cell therapy response in the early phase following STEMI. However, the use of both placebo and cell-treated subjects was an important step to validate the findings. The leads generated in the cell-treated cohort were not implicated in the placebo-treated group. Other targets for future panels include osteopontin^40,41^, exosomes, connexin 43^42^, and novel small molecules including miRNA.

We observed that the patients who improved after placebo also had biomarkers that correlated with outcome. Some of these biomarkers were also seen in the BM-MNC-treated cohort including Eotaxin and MIP1a, which suggests that these improvements might occur regardless of cell therapy. However, IL-15 and SCF were the two lead candidates from the BM-MNC-treated group that had no correlation in the placebo subjects. Thus, elevated levels of these markers may have a specific role in increasing the efficacy of autologous BM-MNC therapy.

Baseline biomarker measurement is a relatively quick and affordable approach to personalized management of patients, which could increase the efficacy of cell therapy via more rational and precise inclusion criteria. This study demonstrated specific cytokines in both BM-MNC and control groups, which led to improved cardiac function measure. The limitations of this study include the relatively low number of subjects leading to small sample bias. Clearly, our findings require confirmation in replication cohorts with larger patient populations. Another limitation is that the TIME and Late-TIME studies did not show aggregate improvements in the cell-treated patients relative to the placebo-treated patients.

## Conclusion

In this study, we identified a set of circulating plasma proteins associated with a favorable response to BM-MNC therapy after STEMI. The identified biomarkers might improve the selection criteria to conduct precision medicine, which personalizes the therapeutic decision and enhances the clinical outcome of STEMI patients undergoing stem cell treatment.

### Data Availability

The datasets generated during and/or analyzed during the current study are available from the corresponding author on reasonable request.

## Electronic supplementary material

Supplementary Information
