# Author Correction: Circulating Biomarkers to Identify Responders in Cardiac Cell therapy

**DOI:** 10.1038/s41598-018-22121-2

**Published:** 2018-03-06

**Authors:** Jesse V. Jokerst, Nicholas Cauwenberghs, Tatiana Kuznetsova, Francois Haddad, Timothy Sweeney, Jiayi Hou, Yael Rosenberg-Hasson, Eric Zhao, Robert Schutt, Roberto Bolli, Jay H. Traverse, Carl J. Pepine, Timothy D. Henry, Ivonne H. Schulman, Lem Moyé, Doris A. Taylor, Phillip C. Yang

**Affiliations:** 1 Department of NanoEngineering, University of California, San Diego, USA; 2 Research Unit of Hypertension and Cardiovascular Epidemiology, Department of Cardiovascular Sciences, University of Leuven, Leuven, Belgium; 3 Department of Medicine, Stanford University, Stanford, USA; 4 Clinical and Translational Research Institute, University of California, San Diego, USA; 5 Houston Methodist Hospital, Houston, USA; 6 School of Medicine, University of Louisville, Louisville, USA; 7 Minneapolis Heart Institute Foundation at Abbott Northwestern Hospital, Minneapolis, USA; 8 University of Florida College of Medicine, Gainesville, USA; 9 Cedars-Sinai Heart Institute, Los Angeles, USA; 10 Interdisciplinary Stem Cell Institute, University of Miami Miller School of Medicine, Miami, USA; 11 University of Texas School of Public Health, University of Texas Health Science Center, Houston, USA; 12 Texas Heart Institute, CHI St. Luke’s Health Baylor College of Medicine Medical Center, Houston, USA

Correction to: *Scientific Reports* 10.1038/s41598-017-04801-7, published online 30 June 2017

In the original version of this Article, there were errors in Affiliations 6, 7, 8, 9, and 10 which were incorrectly listed as ‘School of Medicine, University of Louisville, Houston, USA’, ‘Minneapolis Heart Institute Foundation at Abbott Northwestern Hospital, Houston, USA’, ‘University of Florida College of Medicine, Houston, USA’, ‘Cedars-Sinai Heart Institute, Houston, USA’, and ‘Interdisciplinary Stem Cell Institute, University of Miami Miller School of Medicine, Houston, USA’ respectively.

The correct affiliations are listed below.

Affiliation 6

School of Medicine, University of Louisville, Louisville, USA.

Affiliation 7

Minneapolis Heart Institute Foundation at Abbott Northwestern Hospital, Minneapolis, USA.

Affiliation 8

University of Florida College of Medicine, Gainesville, USA.

Affiliation 9

Cedars-Sinai Heart Institute, Los Angeles, USA.

Affiliation 10

Interdisciplinary Stem Cell Institute, University of Miami Miller School of Medicine, Miami, USA.

These errors have now been corrected in the PDF and HTML versions of the Article.

