# Supplementary material for: Circulating Biomarkers to Identify Responders in Cardiac Cell therapy
Source: Sci Rep. 2017 Jun 30;7:4419. doi: 10.1038/s41598-017-04801-7 (PMC5493650; doi:10.1038/s41598-017-04801-7)
Supplement: Supplementary file 1 — Supplementary Information [file 41598_2017_4801_MOESM1_ESM.pdf]

Circulating Biomarkers to Identify Responders in Cardiac Cell therapy

Jesse V. Jokerst[1], Nicholas Cauwenberghs[2], Tatiana Kouznetsova[2], Francois Haddad[3], Timothy Sweeney[3], Jiayi Hou[4], Yael Rosenberg-Hasson[3], Eric Zhao[1], Robert Schutt[5], Roberto Bolli[6], Jay H. Traverse[7], Carl J. Pepine[8], Timothy D. Henry[9], Ivonne Schulman[10], Lem Moyé[11], Doris A. Taylor[12], and Phillip C. Yang[3]

[1]Department of NanoEngineering, University of California, San Diego

[2]Research Unit of Hypertension and Cardiovascular Epidemiology, Department of Cardiovascular Sciences, University of Leuven,

[3]Department of Medicine, Stanford University,

[4]Clinical and Translational Research Institute, University of California, San Diego

[5]Houston Methodist Hospital,

[6]School of Medicine, University of Louisville,

[7]Minneapolis Heart Institute Foundation at Abbott Northwestern Hospital,

[8]University of Florida College of Medicine,

[9]Cedars-Sinai Heart Institute,

[10]Interdisciplinary Stem Cell Institute, University of Miami Miller School of Medicine,

[11]University of Texas School of Public Health, University of Texas Health Science Center – Houston, and

[12]Texas Heart Institute, CHI St. Luke's Health Baylor College of Medicine Medical Center

Corresponding Author:
Jesse Jokerst
9500 Gilman Drive
La Jolla, California 92093-0448
P-858-246-0896, F- 858-534-9553
jjokerst@ucsd.edu

Subject codes: biomarkers, cell therapy, myocardial infarction

SUPPLEMENTARY METHODS

Enrollment and Specimens. Banked plasma samples were obtained from the TIME[1,2] (clinicaltrials.gov no. NCT00684021) and Late-TIME trials[3] (clinicaltrials.gov No. NCT00684060), which were conducted between July 2008 and November 2011. Briefly, these trials assessed left ventricular function at baseline and six months after intracoronary delivery of autologous BMC following anterior STEMI and reperfusion with primary PCI. BMC administration was performed 3-7 days (TIME) or 14-21 days (Late-TIME) after reperfusion. The primary inclusion criterion was LVEF <45% as assessed by echocardiography persisting 1-2 days after reperfusion. Patients with previous bypass surgery or prior STEMI with residual left ventricular dysfunction were excluded. The cell harvesting, cell processing, and outcome collection were identical in both trials. Blood samples were collected after reperfusion but prior to administration of cells in tubes containing EDTA as an anticoagulant. The samples were centrifuged, and the plasma was decanted and stored at -80° C. All samples were collected after subjects provided written informed consent. This study received Institutional Review Board approval at each center.

Power Calculation. We performed multiple power calculations[4] to estimate the number of samples needed for significance using R software[5]. The lowest number of subjects needed to achieve 80% power for the different analyses was 69 for ANOVA, 53 for multivariate regression with two continuous and two categorical variables, 47 for a continuous correlation, and 78 for a simple t-test. Thus, all of our analyses were well-powered at 80%, except for t-test with the wall motion outcomes (77 samples or ~79% power).

Data Collection. Banked plasma samples were available from 82 BMC-treated patients (TIME n=33 and Late-TIME n=49) and 66 placebo-treated (TIME n=40 and Late-TIME n=26). Outcome data were collected at baseline and six months after therapy. The key outcome data indicating a favorable response to stem cell therapy were changes in: LVEF (ΔEF, increase), end diastolic volume index (ΔEDVI, decrease), end systolic volume index (ΔESVI, decrease), infarct zone wall motion (ΔINZ, increase), and border zone wall motion (ΔBDZ, increase) as measured by MRI. After excluding 5 BMC-treated subjects with incomplete outcome data, we had 78 subjects with ΔEF, ΔEDVI, and ΔESVI, and 77 subjects with ΔINZ and ΔBDZ outcome data. Five placebo subjects were also excluded due to incomplete outcome data.

All plasma samples were analyzed for 63 circulating proteins via Luminex assay (Luminex 200, Austin, TX) as described previously[6]. These biomarkers (**Fig. 1**) were chosen based on their role in regenerative medicine including SCF, SDF1, and colony stimulating factors[7]. We also included biomarkers of growth (e.g. VEGF)[8], repair (TNFα)[8], adhesion (VCAM1)[9], and inflammation (interleukins and chemokines). Reagents (eBioscience, San Diego, CA) were used according to the manufacturer's instructions. Samples were mixed with beads for 2 h at room temperature and overnight at 4 ^{o}C. After washing, the beads were reacted with biotinylated detection antibody for 2 h followed by another wash and streptavidin-fluorophore. Data were recorded as median fluorescent intensity (MFI) for at least 100 bead counts for each sample and each biomarker. We validated the assays using standard curves and calibration samples for every biomarker according to standard Luminex protocols[10-12]. All samples were read in duplicate and the average MFI was used for analysis.

SUPPLEMENTARY DATA

Table S1. Selected cytokines related to absolute and relative change in LV indexes as identified by PLS analysis (VIP > 1.5).

LV change after therapy	Positive coefficients	Negative coefficients
ΔEF		
Absolute Δ	IL15, IL5, SCF	
Relative Δ	IL15, IL5, MIP1B, PDGFBB	
ΔEDVI		
Absolute Δ		BDNF, ENA78, SCF, MCP1, PAI1, PDGFBB, IL27, IL12p70
Relative Δ		BDNF, ENA78, SCF, MCP1, PAI1, IL27, IL12p70, MCSF, IL13
ΔESVI		
Absolute Δ		PDGFBB, MCP1, IL12p70, SCF, IL5, IL13, BDNF, IL23, IL27, IL9
Relative Δ	RESISTIN	PDGFBB, IL12p70, SCF, IL5, IL13, IL27, INFG, VEGFD, PAI1, IL15
ΔINZ		
Absolute Δ	IL15, FASL, IL17A, IL31, IL4, FGFB, TNFB, IL9, IL23	
Relative Δ	VCAM1, IL27, VEGFD, SCF, IL15, EGF, IL4	MCP1
ΔBDZ		
Absolute Δ	IL15, IL9, IL5, IL4	
Relative Δ	IL1A, TRAIL, IL21, VEGF, MIG, MIP1B, SDF1A, IL17F, MIP1A, TGFA, IFNB	

Table S2. Selected cytokines related to response after cell therapy as identified by PLS analysis (VIP > 1.5).

Response to cell therapy	Positive coefficients	Negative coefficients
ΔEF > 0% (n=49)	IL15, INFG, SCF	
ΔEDVI < 0 (n=33)	MCP1, SCF, IL12P70, MCSF, IL27, IL13	IL12P40, IL31, GMCSF
ΔESVI < 0 (n=42)	SCF, MCP1, IL12p70, LIF, IL5, PAI1, IL27, IL7, TGFB	RESISTIN
ΔINZ > 0 (n=43)	IL9, FASL, FGFB, TNFB, IL8, IL31, IL17A, IL4, GROA	
ΔBDZ > 0 (n=44)	IL12p40, IL9, IL15, EOTAXIN, NGF, FASL	
ΔEF > 0% + ΔEDVI < 0 + ΔESVI < 0 (n=18)	SCF, IL12p70, IL27, MCSF, BDNF, IL13, PDGFBB, VCAM1, IFNG	

Table S3. Selected cytokines related to absolute change in LV indexes after placebo treatment as identified by PLS analysis (VIP > 1.5).

LV index	Positive coefficients	Negative coefficients
Δ EF	IL22, FASL, IL23, MCP1, IL9, MCSF, IL12P40, IL1B	HGF, IL1RA
Δ EDVI	IL1RA	MIP1A, IL1A, VEGF, SDF1A, TRAIL, IL21, IL23, MCP1, MIP1B, MIG
Δ ESVI	HGF, IL1RA	IL23, MCSF, FASL, IL1A, IL9, MCP1, MIP1A, IL31, TRAIL, VEGF
Δ INZ	IL22	Resistin, IL1B, BDNF, HGF, ENA78, IL12P70
Δ BDZ	MCP1, IL18, Eotaxin, GMCSF, Leptin, GMCSF, Leptin, IL1B, IL22	HGF

Table S4. Selected cytokines related to response after placebo treatment as identified by PLS analysis (VIP > 1.5).

Response to cell therapy	Positive coefficients	Negative coefficients
Δ EF > 0% (n=49)	None	HGF, LIF, IL1RA, ENA78, IL1B, IL17A, TGFB, MIG
Δ EDVI < 0 (n=33)	MIP1A, SDF1A, VEGF, MCP1, CD40L, IL21	Leptin
Δ ESVI < 0 (n=42)	IL18, MIP1A, IL1A	IL15, HGF, IL1RA, GMCSF
Δ INZ > 0 (n=43)	VEGFD	IL1B, Resistin, BDNF, ENA78, IL17A, IL12P70, TGFB, HGF
Δ BDZ > 0 (n=44)	IL12P70, VEGFD, SCF, MCP1, IL17A, IL9	None
Δ EF > 0% + Δ EDVI < 0 + Δ ESVI < 0 (n=18)	CD40L, Eotaxin, IL5	LIF, IL7

References

1 Traverse, J. H. *et al.* Effect of the use and timing of bone marrow mononuclear cell delivery on left ventricular function after acute myocardial infarction: the TIME randomized trial. *Jama* **308**, 2380-2389 (2012).

2 Traverse, J. H. *et al.* Rationale and design for TIME: A phase II, randomized, double-blind, placebo-controlled pilot trial evaluating the safety and effect of timing of administration of bone marrow mononuclear cells after acute myocardial infarction. *American Heart Journal* **158**, 356-363, doi:http://dx.doi.org/10.1016/j.ahj.2009.06.009 (2009).

3 Traverse, J. H. *et al.* Effect of intracoronary delivery of autologous bone marrow mononuclear cells 2 to 3 weeks following acute myocardial infarction on left ventricular function: the LateTIME randomized trial. *JAMA* **306**, 2110-2119 (2011).

4 Cohen, J. *Statistical power analysis for the behavioral sciences*. (Academic press, 2013).

5 Knezevic, S. Z., Streibig, J. C. & Ritz, C. Utilizing R software package for dose-response studies: the concept and data analysis. (2009).

6 Xue, J. *et al.* Alternatively activated macrophages promote pancreatic fibrosis in chronic pancreatitis. *Nature communications* **6** (2015).

7 Zohlnhöfer, D. *et al.* Stem cell mobilization by granulocyte colony-stimulating factor in patients with acute myocardial infarction: a randomized controlled trial. *Jama* **295**, 1003-1010 (2006).

8 Taichman, R. S. Blood and bone: two tissues whose fates are intertwined to create the hematopoietic stem-cell niche. *Blood* **105**, 2631-2639 (2005).

9 Whetton, A. D. & Graham, G. J. Homing and mobilization in the stem cell niche. *Trends in cell biology* **9**, 233-238 (1999).

10 Rosenberg-Hasson, Y., Hansmann, L., Liedtke, M., Herschmann, I. & Maecker, H. T. Effects of serum and plasma matrices on multiplex immunoassays. *Immunologic research* **58**, 224-233 (2014).

11 Dunbar, S. A. Applications of Luminex® xMAP™ technology for rapid, high-throughput multiplexed nucleic acid detection. *Clinica Chimica Acta* **363**, 71-82 (2006).

12 Haddad, F. *et al.* Immunologic Network and Response to Intramyocardial CD34+ Stem Cell Therapy in Patients With Dilated Cardiomyopathy. *Journal of cardiac failure* **21**, 572-582, doi:10.1016/j.cardfail.2015.03.011 (2015).
